# White Adipose Tissue Browning in the R6/2 Mouse Model of Huntington’s Disease

**DOI:** 10.1371/journal.pone.0159870

**Published:** 2016-08-03

**Authors:** Andrew C. McCourt, Lovisa Jakobsson, Sara Larsson, Cecilia Holm, Sarah Piel, Eskil Elmér, Maria Björkqvist

**Affiliations:** 1 Brain Disease Biomarker Unit, Department of Experimental Medical Science, Lund University, BMC A10, 22184 Lund, Sweden; 2 Section for Diabetes, Metabolism and Endocrinology, Department of Experimental Medical Science, Lund University, BMC C11, 221 84 Lund, Sweden; 3 Mitochondrial Medicine, Department of Clinical Sciences, Lund University, BMC A13, 221 84 Lund, Sweden; University of Minnesota Twin Cities, UNITED STATES

## Abstract

Huntington’s disease (HD) is a fatal, autosomal dominantly inherited neurodegenerative disorder, characterised not only by progressive cognitive, motor and psychiatric impairments, but also of peripheral pathology. In both human HD and in mouse models of HD there is evidence of increased energy expenditure and weight loss, alongside altered body composition. Unlike white adipose tissue (WAT), brown adipose tissue (BAT), as well as brown-like cells within WAT, expresses the mitochondrial protein, uncoupling protein 1 (UCP1). UCP1 enables dissociation of cellular respiration from ATP utilization, resulting in the release of stored energy as heat. Hyperplasia of brown/beige cells in WAT has been suggested to enhance energy expenditure. In this study, we therefore investigated the gene expression profile, histological appearance, response to cold challenge and functional aspects of WAT in the R6/2 HD mouse model and selected WAT gene expression in the full-length Q175 mouse model of HD. WAT from R6/2 mice contained significantly more brown-like adipocyte regions and had a gene profile suggestive of the presence of brown-like adipocytes, such as higher *Ucp1* expression. Cold exposure induced *Ucp1* expression in R6/2 inguinal WAT to a markedly higher degree as compared to the thermogenic response in WT WAT. Alongside this, gene expression of transcription factors (*Zfp516* and *Pparα*), important inducers of WAT browning, were increased in R6/2 inguinal WAT, and *Creb1* was highlighted as a key transcription factor in HD. In addition to increased WAT *Ucp1* expression, a trend towards increased mitochondrial oxygen consumption due to enhanced uncoupling activity was found in inguinal R6/2 WAT. Key gene expressional changes (increased expression of (*Zfp516* and *Pparα*)) were replicated in inguinal WAT obtained from Q175 mice. In summary, for the first time, we here show that HD mouse WAT undergoes a process of browning, resulting in molecular and functional alterations that may contribute to the weight loss and altered metabolism observed with disease progression.

## Introduction

Huntington’s disease (HD) is a devastating autosomal dominant neurological disorder characterised by classical neurological symptoms, such as progressive cognitive, motor and psychiatric impairments [1]. The causal mutant gene (created by an expanded polyglutamine tract in exon 1 of the huntingtin gene) and the encoded protein are however, ubiquitously expressed throughout the body [2, 3] and HD central pathology is accompanied by complex peripheral pathology, including skeletal muscle atrophy, altered body composition and progressive weight loss [4–6].

Progressive weight loss is a feature of both human HD [7–10] and of HD mouse models [11]. Despite this, the cause of HD weight loss is not fully known. Noteworthy, is that HD weight loss has been shown to be linked to the number of CAG repeats in the mutated gene [12]. Studies have indicated that body weight loss results from altered metabolism [10, 13], such as increased oxygen consumption [11].

Several mouse models of HD exist, and studies have demonstrated progressive alterations in white adipose tissue (WAT) mass with age in both the transgenic R6/2 mouse model and in full length CAG140 knock-in HD mice, and that these changes are accompanied by an impairment in the expression of mature adipocyte genes [14, 15]. Altered levels of adipose tissue derived leptin and adiponectin have been demonstrated [15], as well as reduced adipose tissue lipolytic function [14]. Further, a direct effect of mutant huntingtin has been demonstrated in adipocytes *in vitro*. Impaired gene expression and lipid accumulation was demonstrated in adipocytes expressing mutant Htt, replicating findings seen in mouse models of disease [15]. Supportive of this, we recently showed an altered gene expression profile in human HD subcutaneous WAT [16].

Two types of adipose tissue can be identified, based on histological appearance and functional aspects. White adipocytes are mainly unilocular, while brown adipocytes displays small multilocular lipid droplets. WAT impacts on whole body energy metabolism both through its role as an energy storage source and via its capacity as an endocrine organ [17]. Brown adipose tissue (BAT), is responsible for energy dissipation through uncoupling, generating heat [18]. Besides classical brown and white adipocytes, knowledge has accumulated over the last decade describing a sub-set of cells within WAT that can be converted to”beige”,”brite” or”brown-in-white” adipocytes [19] (in this study referred to as brown-like adipocytes), sharing many of the morphological and functional features of brown adipocytes. These cells can upon stimulation, such as cold exposure, and under the influence of certain transcription factors, adopt brown-like features, such as *Ucp1* expression and multilocular lipid droplets [20, 21].

Given previously published data that the R6/2 HD mouse model exhibits adipose tissue abnormalities [14, 15] in combination with weight loss and increased energy expenditure [11], we here investigated whether HD mouse WAT exhibits brown features and whether this results in altered WAT function.

## Materials & Methods

### Animals

#### R6/2 mice

Twelve-week old male R6/2 and wild type (WT) littermate mice were used for R6/2 experimental procedures. Transgenic mice of the R6/2 line and their WT littermates were obtained by crossing heterozygous males with females of their background strain (C57BL/6).

#### Q175 mice

Twelve- and eighteen-month old male Q175 and WT mice were utilised for Q175 experimental procedures. As with the R6/2 colony, full-length Q175/175 knock-in mice and their WT littermates were obtained by crossing heterozygous males with females of their background strain (C57BL/6).

Polyglutamine (CAG) repeat length was determined by polymerase chain reaction (PCR) assay [22]. CAG repeat lengths were in the range of 280–315 for the R6/2 mice and 195–230 for the Q175 mice.

All mice were obtained by in house breeding and housed in littermate-groups with food and water available *ad libitum*, under standard conditions (12:12 h light:dark cycle, 22°C). No mice became ill and reached ethical endpoints, nor did any mice receive medical treatment prior to time of experimental endpoint. The experimental endpoint here used, was mid-stage disease, i.e. prior to overt HD related symptom. Experimental procedures were in accordance with Swedish legislation and approved by the Regional Ethical Committee of Malmö/Lund, Sweden.

### Adipose tissue collection

Mice were euthanized at different time points by cervical dislocation before immediate tissue collection. Subcutaneous inguinal white, intra-abdominal epididymal white and inter-scapular brown adipose tissues were immediately dissected, snap-frozen in liquid nitrogen, and stored at -80°C until analyzed.

### Histology

WAT from two depots (subcutaneous inguinal and epididymal) and interscapular BAT from 12-week old male R6/2 and littermate WT were dissected immediately following euthanization and placed into 4% paraformaldehyde overnight. Tissues were then transferred to 70% ethanol solution and stored at 4°C for up to one week before paraffin imbedding. After paraffin embedding, tissues were then cut into 7 μm thick sections, mounted and then stained with haematoxylin and eosin.

Slides were then examined by light microscopy (Olympus BX53, Olympus, Tokyo, Japan): 100 cells from each mouse were selected for area measurements at 20x magnification. Digital images were acquired using a digital camera (Olympus DP73, Olympus, Tokyo, Japan) and cell areas were measured using cellSens Dimensions 1.11 software (Olympus, Tokyo, Japan). Slides were coded to allow for blinded measuring. Mean adipocyte areas for each of the two groups, R6/2 and WT, were then compared.

### RNA extraction

Total RNA was extracted from inguinal white and interscapular brown adipose tissue using the RNeasy Lipid Tissue Mini Kit (Qiagen GmbH, Hilden, Germany) following manufacturer’s protocol. RNA integrity was analysed by Agilent 2100 Bioanalyzer (Agilent Technologies, CA, USA), and only samples with RIN values greater than or equal to 6.50 were utilised for affymetrix analysis. For RT-qPCR validations, RNA was then reverse transcribed to cDNA using iScript™ cDNA Synthesis Kit (BioRad, CA, USA) and stored at -20°C.

### Affymetrix

Gene expression analysis was performed on samples with RIN values greater than or equal to 6.50, using Affymetrix GeneChip® Mouse Gene 2.0 ST Array and RT-qPCR. The affymetrix data discussed in this publication have been deposited in NCBI’s Gene Expression Omnibus [23] and are accessible through GEO Series accession number GSE79711 (http://www.ncbi.nlm.nih.gov/geo/query/acc.cgi?acc=GSE79711).

### Microarray Data Analysis

Microarray data were initially pre-processed and normalized using Robust Multi-array Analysis (RMA) method [24]. These analyses were performed using Affymetrix Expression Console Software v1.1.2. Non-annotated probe sets and probe sets not having signal intensity above the median of negative control intensity signals in each group were excluded. Replicate probe sets were merged by the median of signal intensity values.

To identify significantly differentially expressed genes between groups, we used Significance Analysis of Microarrays (SAM) method [25]. SAM analysis was performed using TMEV v4.0 software.

We selected differentially expressed genes having q-value < 10% for the Pathway analysis, which was performed using MetaCore™ pathway analysis software [26] (see https://portal.genego.com/help/P-value_calculations.pdf for detailed explanation of analyses).

### Targeted microarray cDNA synthesis & RT-qPCR

500 ng total RNA from inguinal white and interscapular brown adipose tissue was reverse-transcribed into cDNA using the RT^2^ PCR array first strand kit (SABiosciences, MD, USA). The cDNA was then mixed with RT^2^ qPCR mastermix containing SYBR Green (SABiosciences, MD, USA), and 25 μl of PCR mixture was aliquoted into each well of the 96-well PCR array plate. The Mouse Adipogenesis RT^2^ ProfilerTM PCR array (SABiosciences, Catalog no. PAMM-049Z) targets 84 genes related to adipogenesis. Quantitative real-time PCR (qPCR) cycles were run on a BioRad CFX96 Touch Real-Time PCR Detection System, and performed according to the manufacturer's instructions.

#### RT-qPCR

Validations were carried out using RT-qPCR on inguinal WAT and iBAT from 12-week R6/2 mice and WT littermates. Further validations were then performed on 12- and 18-month Q175 mice and WT littermates. For RT-qPCR experiments, all samples were run in triplicate for each target gene and housekeeping gene, and relevant negative and positive controls were run on each plate. Melt curves were inspected for all assays, with the Tm checked to be within known specifications for each assay. Sample assay data points were included in data analysis only if detected with Ct < 37 and at least 3 Ct values lower than the corresponding negative control [27]. Any data that did not pass these criteria were omitted from all further analyses. Primers utilised for RT-qPCR validations (Table 1) were designed using QuantPrime [28] or PrimerQuest from Integrated DNA Technologies (http://eu.idtdna.com/PrimerQuest).

**Table 1:** Primer sequences.

Target	Forward primer	Reverse primer
***Cd137***	**AGGAGCTAACGAAGCAGGGTTG**	**TCCCGGTCTTAAGCACAGACCTTC**
***Cebpβ***	**CGCGACAAGGCCAAGAT**	**GCTGCTCCACCTTCTTCTG**
***Cfd***	**CATGAACCGGACAACCTGCAATCT**	**ACGTAACCACACCTTCGACTGCAT**
***Creb1***	**GAGAGCTGGTATGTCAGGAATG**	**CCAGAAGAGATGCAGGAGAAAG**
***Gata3***	**AAGGCACGATCCAGCACAGAAG**	**TTATGGTAGAGTCCGCAGGCATTG**
Gusb*	**CCGACTTCATGACGAACCAGTCAC**	**TGTCTCTGGCGAGTGAAGATCC**
Hsp90ab1*	**ATGATTAAACTAGGCCTGGGCATC**	**GCTTTAATCCACCTCTTCCATGCG**
***Jun***	**CGCTGGAAAGCAGACACTTTGGTT**	**TCCATGGGTCCCTGCTTTGAGAAT**
***Pgc1α***	**CGTAAATCTGCGGGATGATGGAG**	**AGCGTCACAGGTGTAACGGTAG**
***Pparα***	**TCAGGGTACCACTACGGAGTTCA**	**AGTTCGCCGAAAGAAGCCCTTAC**
***Pparγ***	**AGGAAAGACAACGGACAAATCACC**	**ATTCGGATGGCCACCTCTTTGC**
***Sirt1***	**AGCAACATCTCATGATTGGCACCG**	**TCTGCCACAGCGTCATATCATCCA**
***Taz***	**GCAGAGAACAAGTCAGCTGTGGAG**	**AGCCGCTGGAATTCCTCTTGAATG**
Tbp*	**TCTGAGAGCTCTGGAATTGTACCG**	**TGATGACTGCAGCAAATCGCTTG**
***Ucp1***	**GCATTCAGAGGCAAATCAGC**	**GCCACACCTCCAGTCATTAAG**
***Wnt5b***	**AGAAAGACGGTTCTGTCACCTGCT**	**AGCATGCCCTGTCATTCTCAAAGC**
***Zfp516***	**CTTTCTGTTGACGGCGTTTG**	**TCCTAACTCTCACCTTCACTCT**

Primer sequences used for RT-qPCR validations. Primers were designed using QuantPrime [28] or PrimerQuest from Integrated DNA Technologies (http://eu.idtdna.com/PrimerQuest).

* denotes housekeeping genes.

### Western blot

For UCP1 protein determination, mitochondrial fractions were extracted from approximately 50 mg of inguinal white and interscapular brown adipose tissue as previously described [29]. Briefly, adipose tissue samples were homogenized in 600 μl of supplemented sucrose buffer containing 250 mM sucrose, 20 mM HEPES (pH 7.4), 1mM EGTA, 1 mM DTT, 1 mM PMSF and protease inhibitor cocktail (Roche, Basel, Switzerland). The homogenates were then centrifuged at 500 x g for 12 min at 4°C in order to pellet the nucleus and cell debris. The supernatant was then centrifuged at 12000 x g for 20 min at 4°C and the supernatant removed. The remaining pellet was then resuspended in 100 μl of supplemented sucrose buffer and the protein concentration was determined by BCA assay (Thermo Scientific, MA, USA).

Proteins were separated by SDS-polyacrylamide gel electrophoresis and then transferred to a PVD membrane using a Trans-Blot Turbo System (Bio-Rad, CA, USA). The membranes were then probed with rabbit anti-UCP1 (1:750; Sigma-Aldrich, MO, USA), followed by washing, and probing with horseradish peroxidase-conjugated secondary antibody (1:5000; Dako, Glostrup, Denmark). Bands were visualized with Western Blotting Luminol Reagent (Santa Cruz, TX, USA) and imaged with the ChemiDoc MP Imaging System (Bio-Rad, CA, USA).

### Cold challenge

For cold challenges, R6/2 and WT littermates were housed individually and exposed to 4°C for 4 hours on two consecutive days. Following the second cold exposure, mice were euthanized by cervical dislocation before immediate inguinal white and interscapular brown adipose tissue collection, as described above.

### Lipolysis

For lipolysis experiments, R6/2 and WT littermates were euthanized by cervical dislocation and intra-abdominal epididymal white adipose tissue was excised. Adipocytes were isolated by collagenase digestion and utilized in experiments immediately. The preparation was performed according to [30] except that the collagenase concentration was lowered from 1 mg/ml to 0.6 mg/ml and the incubation time was prolonged from 30 min to 75 min. Cells were diluted to a concentration of 5% in Krebs Ringer buffer containing 25 mM HEPES, 1% fatty acid-free BSA, 200 nM adenosine and 2 mM glucose. Lipolysis was modulated by the addition of 20 nM isoprenaline. Cells were incubated at 37°C in a shaking incubator, 150 rpm, for 30 min. Experiments were ended by incubating the cells on ice for 30 min before a 150 μl aliquot of the incubation media was taken for analysis of glycerol release. Glycerol concentration was determined using a commercially available kit with the addition of Amplex Ultra Red, a hydrogen peroxide sensitive fluorescence dye, as described by Clark et al. [31].

### Oxygen consumption

High-resolution respirometry was employed to measure oxygen consumption of adipocytes (Oxygraph-2k, Oroboros Instruments, Innsbruck, Austria). Subcutaneous inguinal WAT was collected from 12 week R6/2 and WT mice and incubated in ice-cold mitochondrial respiration medium (MiR05) [32]. Pieces of tissue (60–70 mg) were weighed directly into 2 mL (MiR05) and homogenised by fine chopping. Respirometry was performed at a stirrer speed of 750 rpm and 37˚C. All respiratory values were corrected for the oxygen solubility factor of the medium (0.92).

For oxygen consumption measurements of adipocytes, endogenous cellular respiration rate was initially recorded, followed by basal_CI+II_ respiratory rate after injection of the complex I and II substrates malate (5mM), pyruvate (5mM), glutamate (5mM) and succinate (10mM). Cells were then permeabilized by addition of digitonin (2μg/mg tissue) to assure complete access of the substrates and exclude that any differences may be caused by differently permeable cell membranes of the WT and R6/2 WAT. Then, ADP was added (1mM) to measure maximal coupled respiration followed by titration with carbonyl cyanide 4-(trifluoromethoxy) phenylhydrazone (FCCP) in 1μM steps to measure maximal uncoupled respiration. Non-mitochondrial oxygen consumption, determined in the presence of 50μg/ml antimycin A, was subtracted from all respiratory states. From the measured parameters, the following ratios were calculated: Basal_CI+II_/FCCP, Maximal coupled/ Basal_CI+II_.

### Statistics

Targeted microarray data: Two-tailed student's t-test was used to calculate P-values for the replicate 2^-ΔCt^ values for each gene in the control group and treatment groups. Data were analyzed by the RT2 Profiler PCR Array Data Analysis software, version 3.5 (http://pcrdataanalysis.sabiosciences.com/pcr/arrayanalysis.php), using the comparative Ct method [33] with normalisation of the raw data to the two housekeeping genes, *Gusb* (glucuronidase β) and *Hsp90ab1* (heat shock protein 90 alpha (cytosolic), class B member 1). A critical value of P < 0.05 was used as a significance threshold for all comparisons.

RT-qPCR: RT-qPCR analysis was performed using Bio-Rad CFX Manager 3.1 software (BioRad). Data were analyzed using the ΔΔCt method and normalized to the housekeeping genes: *Gusb; Hsp90ab1*; and TATA Box Binding Protein (*Tbp*). Two-tailed student’s t-test was used for comparisons in gene expression levels and a critical value of P < 0.05 was used as a significance threshold.

Western blots: Two-tailed student’s t-test was used for statistical analysis of densitometric data, with P < 0.05 considered statistically significant.

Cold challenge/lipolysis: Two-way ANOVA, followed by Tukey’s post-hoc analysis was used for comparisons between groups, with a critical value of P < 0.05 used as a significance threshold.

Oxygen consumption: Two-tailed student’s t-test was used for statistical analysis of respiration data between the two groups, with P < 0.05 considered statistically significant.

Correlation: Two-tailed Pearson correlation analysis was used to test associations between *Ucp1* expression and mouse weight, with P < 0.05 considered statistically significant.

## Results

### R6/2 mouse WAT displays histological features suggesting presence of brown-like adipocytes

**Fig 1:**
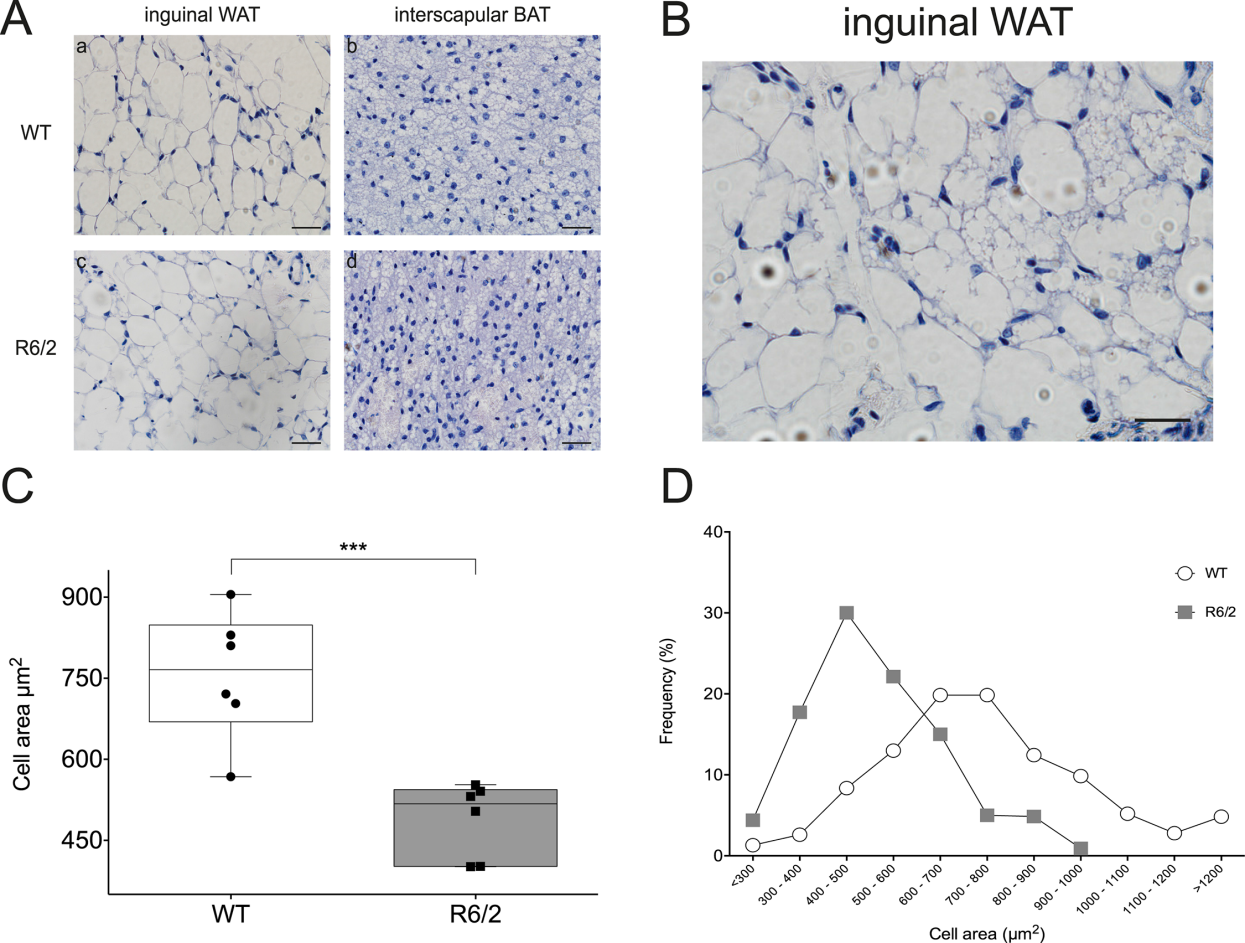
Brown-like adipose features within WAT in R6/2 mice. **A** Representative images of inguinal white adipose tissue (WAT) and interscapular brown adipose tissue (iBAT) from wild type (WT) and R6/2 transgenic mice. Scale bars = 20 μm. WAT is comprised of white adipocytes whose primary function is energy storage. As can be seen in subpanels **a** and **c**, white adipocytes are unilocular, with each one containing a single large lipid droplet that fills the cell. BAT is comprised of brown adipocytes and plays a role in energy metabolism and non-shivering thermogenesis in mammals. As shown in subpanels **b** and **d**, brown adipocytes are smaller than white adipocytes, multilocular, and rich in mitochondria. **B** Representative image from R6/2 inguinal WAT showing signs of small brown-like adipocytes within the tissue. Numerous regions of multilocular adipocytes, reminiscent of brown adipocytes observed in panel **A**, subpanels **b** and **d**, can be found interspersed between large unilocular white adipocytes. Scale bar = 20 μm. **C** Boxplot depicting mean subcutaneous inguinal white adipocyte areas from R6/2 mice (grey) and wild type (WT; white) littermates, n = 6/group, 100 cells/mouse measured. R6/2 mice have significantly smaller inguinal adipocytes than wild type littermates. Circles represent mean cell size for each WT mouse while squares represent mean cell size for each R6/2 mouse. Data presented as mean ± SEM. *** P < 0,001. **D** Cell areas were measured using cellSens software, based on 100 cells per mouse (n = 6/group). Data shows cell size distribution for inguinal WAT of the R6/2 and WT mice. WT mice tend to have a greater quantity of large (> 800 μm^2^) with only a low quantity of small adipocytes (< 300 μm^2^), while the converse is true for R6/2 mice. White circles represent WT mice while grey squares represent R6/2 mice.

Two types of adipose tissue can be identified, based on histological appearance and functional aspects. While white adipocytes are mainly unilocular, brown adipocytes display small multilocular lipid droplets. In addition, brown-like adipocytes may appear within white adipose tissue (WAT) depots, sharing many of the morphological features of brown adipocytes. Therefore, to determine whether any morphological differences existed between R6/2 mice and wild type (WT) adipose tissue, we examined haematoxylin and eosin stained adipose sections taken from subcutaneous white (inguinal) and brown (inter-scapular) adipose tissue (AT) depots from mice of both genotypes (n = 6/genotype/depot) (Fig 1A). We observed brown-like adipose tissue, as determined by presence of multilocular adipocytes, in discrete locations within subcutaneous WAT depots obtained from R6/2 mice (Fig 1B). Importantly, we failed to observe this phenomenon in the WT samples. Cell areas (100 total per mouse: 5 sections per mouse, 20 cells per section) from the subcutaneous inguinal depot of each mouse were quantified and we found R6/2 mice to have significantly smaller adipocytes than WT mice, as reflected by a shift in the curve to the left, with mean cell areas of 488,8 ± 28,32 μm^2^ versus 756,2 ± 48,29 μm^2^ (n = 6/group, P = 0,0007) (Fig 1C and 1D). Similar histological appearance of multilocular adipocytes could also be found in R6/2 epididymal WAT (see S1 Fig).

### Gene expression changes in R6/2 WAT

**Fig 2:**
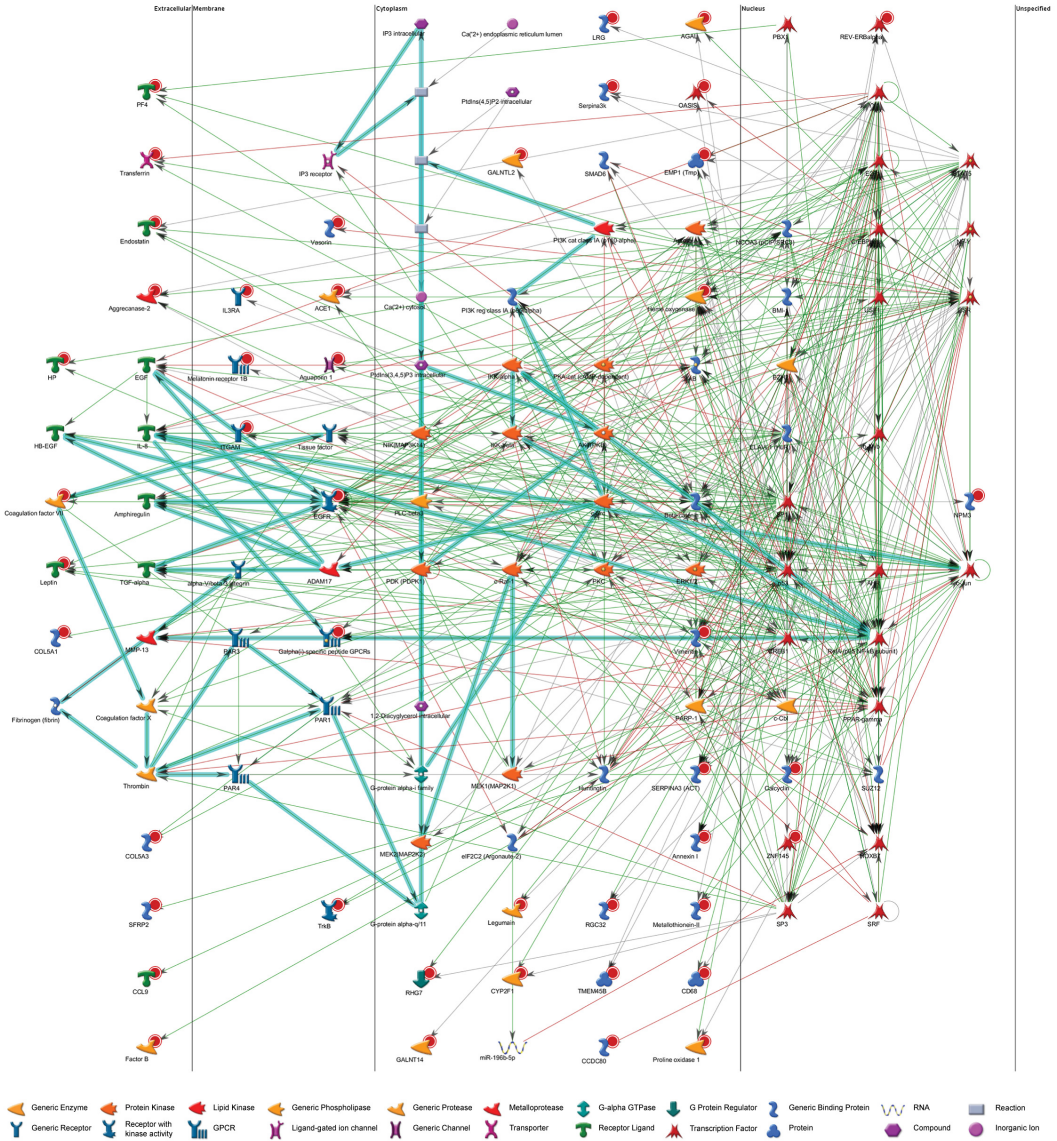
Network analysis. Network analysis based on the top 69 significant results from inguinal WAT of wild type (WT) vs. R6/2 mouse (n = 6/group) affymetrix analysis with each target organised by cellular location. Red circles indicate downregulation in R6/2 mice relative to WT controls from our dataset. Green lined arrows indicate positive interaction/activation; red lines, negative interaction/inhibition; grey lines, unspecified interaction.

The observed histological alterations suggest that in R6/2 mice, WAT may be undergoing a browning phenomenon. To further investigate this, we set out to study whether alterations in subcutaneous WAT gene expression exist in R6/2 mice versus WT. We performed affymetrix analysis on RNA extracted from subcutaneous inguinal WAT from R6/2 and WT mice (n = 6/group). SAM analysis was performed to identify differentially expressed genes between the two genotypes. Setting thresholds for fold change and false discovery rate (1,5 fold and 5%, respectively) we found a total of 69 significantly downregulated genes in R6/2 mice as compared with WT (see S1 Table for full dataset). Notably, we observed highly significant downregulation of haptoglobin (*Hp*, downregulated 4.6 fold), a gene highly expressed in white adipose tissue and lysyl oxidase (*Lox*, downregulated 2.0 fold), a positive regulator of white preadipocyte commitment in rodents. Additionally, we observed an upregulation of *Ucp1* (3.0 fold increase). Network and transcription factor analyses based on this dataset using MetaCore™ software were undertaken in order to gain further insight into which factors might facilitate regulation of gene expression in R6/2 subcutaneous WAT. Transcription factor analysis revealed several key transcription factors involved in the browning of white adipose tissue to be highly significant based on our dataset, including specificity protein 1 transcription factor (SP1), cAMP-response element-binding protein 1 (CREB1), CCAAT/enhancer binding protein beta (C/EBPβ) and peroxisome proliferator-activated receptor gamma (PPARγ) (Fig 2; Table 2; S2 Table). Interestingly, our affymetrix results highlighted several altered genes that directly interact with the huntingtin protein (Table 3). Enrichment by pathway maps analysis highlighted Development_BMP7 in brown adipocyte differentiation as highly significant (Table 4; S3 Table).

**Table 2:** Top 15 Transcription factor networks.

Rank	Network	Total nodes	p-value	zScore
**1**	SP1	9	1,760E-28	155,4
**2**	GCR-alpha	8	6,990E-25	144,22
**3**	c-Myc	7	2,620E-21	132,15
**4**	p53	6	9,220E-18	118,95
**5**	C/EBPalpha	6	9,220E-18	118,95
**6**	CREB1	6	9,220E-18	118,95
**7**	ESR1 (nuclear)	5	3,030E-14	104,24
**8**	VDR	4	9,170E-11	87,4
**9**	COUP-TFI	4	9,170E-11	87,4
**10**	PR (nuclear)	4	9,170E-11	155,4
**11**	HIF1A	4	9,170E-11	87,4
**12**	C/EBPbeta	4	9,170E-11	87,4
**13**	PPAR-gamma	4	9,170E-11	87,4
**14**	GATA-3	4	9,170E-11	87,4
**15**	NFIA	4	9,170E-11	87,4

Top 15 transcription factor networks involving the 69 most significantly altered genes from affymetrix analysis of R6/2 WAT versus WT inguinal WAT (see S2 Table for further information).

**Table 3:** Altered genes that directly interact with huntingtin.

From	To	Direction	Effect	Mechanism
**AKT(PKB)**	Huntingtin	Incoming	Activation	Phosphorylation
**CREB1**	Huntingtin	Incoming	Unspecified	Transcription regulation
Huntingtin	**CREB1**	Outgoing	Inhibition	Binding
**E2F1**	Huntingtin	Incoming	Unspecified	Transcription regulation
**EGFR**	Huntingtin	Incoming	Activation	Binding
**eIF2C2 (Argonaute-2)**	Huntingtin	Incoming	Inhibition	Cleavage
Huntingtin	**eIF2C2 (Argonaute-2)**	Outgoing	Unspecified	Binding
Huntingtin	**EZH2**	Outgoing	Activation	Binding
**IKK-beta**	Huntingtin	Incoming	Inhibition	Phosphorylation
**p53**	Huntingtin	Incoming	Activation	Transcription regulation
Huntingtin	**p53**	Outgoing	Inhibition	Binding
**SP1**	Huntingtin	Incoming	Activation	Transcription regulation
Huntingtin	**SP1**	Outgoing	Inhibition	Binding
Huntingtin	**SUZ12**	Outgoing	Activation	Binding
**Thrombin**	Huntingtin	Incoming	Unspecified	Cleavage

Significantly altered gene targets following affymetrix analysis of R6/2 versus WT inguinal WAT that directly interact with huntingtin.

**Table 4:** Top 10 Pathway maps.

Rank	Maps	Total nodes	Nodes in data	p-value	Min FDR
**1**	Immune response_Alternative complement pathway	39	4	3,009E-05	6,078E-03
**2**	O-glycan biosynthesis	60	4	1,661E-04	1,271E-02
**3**	O-glycan biosynthesis / Human version	62	4	1,888E-04	1,271E-02
**4**	Development_BMP7 in brown adipocyte differentiation	39	3	7,706E-04	3,892E-02
**5**	Action of GSK3 beta in bipolar disorder	23	2	5,062E-03	1,914E-01
**6**	Transcription_Role of Akt in hypoxia induced HIF1 activation	27	2	6,940E-03	1,914E-01
**7**	Development_Regulation of CDK5 in CNS	28	2	7,452E-03	1,914E-01
**8**	Colorectal cancer (general schema)	30	2	8,526E-03	1,914E-01
**9**	Action of lithium on synaptic transmission and autophagy	35	2	1,149E-02	1,914E-01
**10**	Development_Lipoxin inhibitory action on PDGF, EGF and LTD4 signaling	36	2	1,213E-02	1,914E-01

Top 10 significant pathway maps based on top 69 significantly altered transcripts between R6/2 and WT inguinal WAT affymetrix analysis (see S3 Table for further information).

Phan and co-workers showed in 2009 that WAT genes involved in differentiation and maturation were altered in HD [15]. Therefore, following affymetrix analysis, we conducted a targeted gene expression study to evaluate the expression of 84 key genes involved in the differentiation and maintenance of mature adipocytes. Analysis of gene expression array data was carried out with a fold regulation cut off set at ± 1.5 and a P-value of 0.05 for statistical significance. With these set limits, in R6/2 mouse inguinal WAT, seven genes showed significant upregulation (Table 5): *Cfd*, Complement factor D (adipsin) (P = 0.034570); *Gata3*, GATA binding protein 3 (P = 0.048637); *Jun*, Jun oncogene (P = 0.013606); *Pparα*, Peroxisome proliferator activated receptor alpha (P = 0.041983); *Sirt1*, Sirtuin 1 (silent mating type information regulation 2, homolog) 1 (S. cerevisiae) (P = 0.005811); *Taz*, Tafazzin (P = 0.014484); *Wnt5b*, Wingless-related MMTV integration site 5B (P = 0.016054). The upregulation of the anti-white gene, *Gata3*, and pro-brown adipocyte gene, *Pparα*, are in line with our affymetrix data showing suggesting upregulation of brown adipose genes (*Ucp1*) and downregulation of white adipose genes (*Hp* and *Lox*). Further, upregulation of *Cfd* suggests possible altered lipolytic function of R6/2 WAT.

**Table 5:** Differential gene expression levels in inguinal white adipose tissue of R6/2 mice.

	Symbol	Gene Name	Fold regulation	*P* value
**Upregulated gene expression**	Adipoq	Adiponectin, C1Q and collagen domain containing	1.56	0.058728
	Agt	Angiotensinogen (serpin peptidase inhibitor, clade A, member 8)	1.59	0.328790
	**Cfd**	**Complement factor D (adipsin)**	**2.47**	**0.034570** *
	Dlk1	Delta-like 1 homolog (Drosophila)	2.29	0.155221
	Fgf10	Fibroblast growth factor 10	1.52	0.137679
	**Gata3**	**GATA binding protein 3**	**1.74**	**0.048637** *
	Hes1	Hairy and enhancer of split 1 (Drosophila)	2.46	0.068451
	Irs1	Insulin receptor substrate 1	1.82	0.081242
	Irs2	Insulin receptor substrate 2	1.54	0.117393
	**Jun**	**Jun oncogene**	**1.55**	**0.013606** *
	Lipe	Lipase, hormone sensitive	1.62	0.098034
	**Pparα**	**Peroxisome proliferator activated receptor alpha**	**2.06**	**0.041983** *
	Ppargc1α	Peroxisome proliferative activated receptor, gamma, coactivator 1 alpha	1.52	0.395472
	Sfrp1	Secreted frizzled-related protein 1	1.70	0.151184
	**Sirt1**	**Sirtuin 1 (silent mating type information regulation 2, homolog) 1 (S. cerevisiae)**	**1.51**	**0.005811** **
	**Taz**	**Tafazzin**	**1.51**	**0.014484** *
	Ucp1	Uncoupling protein 1 (mitochondrial, proton carrier)	1.51	0.990320
	**Wnt5b**	**Wingless-related MMTV integration site 5B**	**1.59**	**0.016054** *
**Down-regulated**	Cdkn1a	Cyclin-dependent kinase inhibitor 1A (P21)	-1.64	0.123238
	Dio2	Deiodinase, iodothyronine, type II	-1.74	0.238260
	Lep	Leptin	-1.73	0.081729

### Gene expression changes support R6/2 WAT brown features

To validate selected gene expression changes observed using affymetrix and targeted array data, we performed RT-qPCR using independently designed primers (see Table 1).

For WAT, we selected all seven significantly altered genes from the targeted array, alongside targets from the affymetrix data, and the BAT marker genes *Ucp1* and *Zfp516*, for RT-qPCR validation.

**Fig 3:**
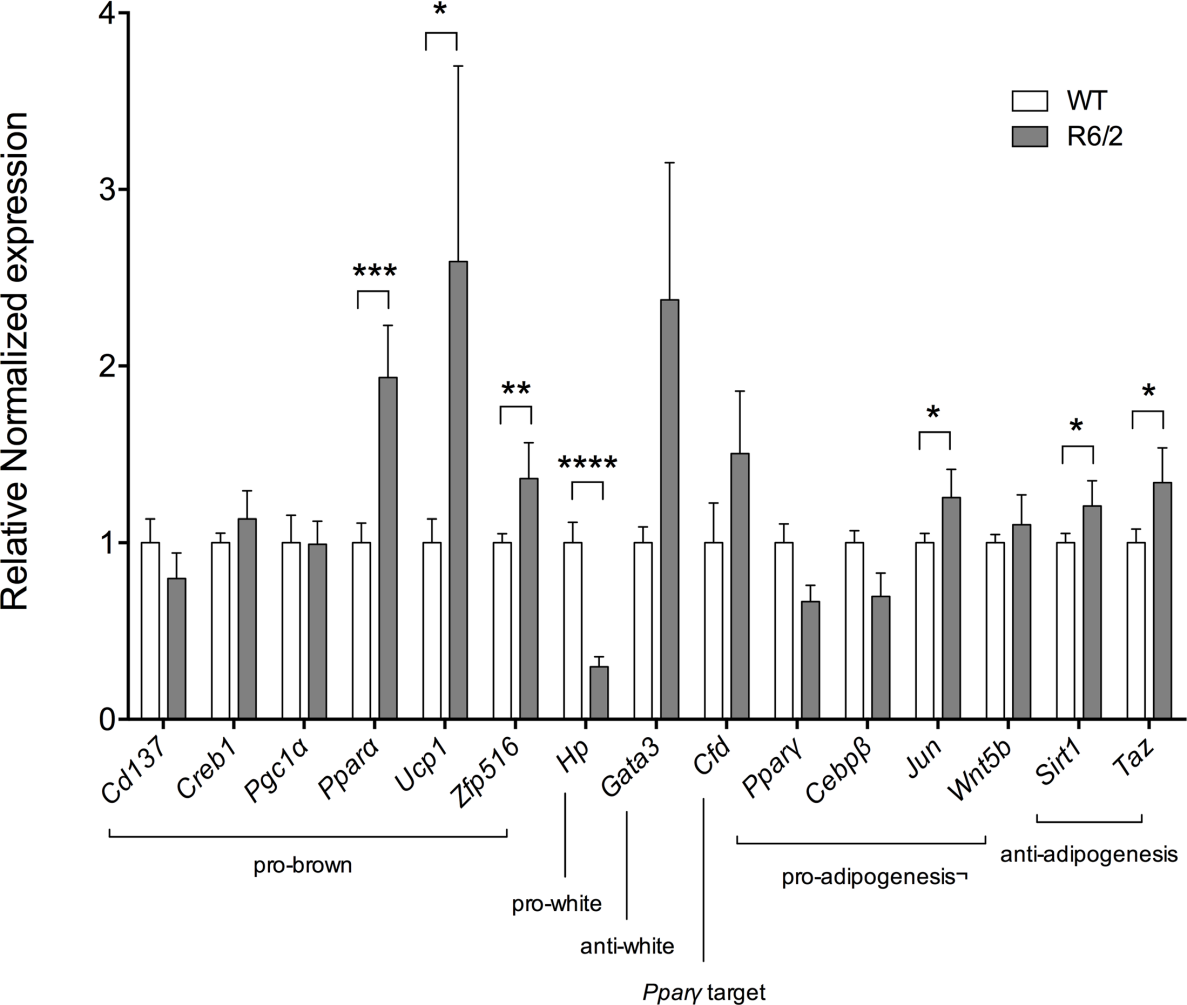
Significantly upregulated pro-brown adipose tissue gene expression in R6/2 mouse inguinal WAT. Normalized gene expression from 12-week R6/2 (grey bars) versus WT (white bars) subcutaneous inguinal WAT (n = 6/group). Here we replicated the upregulated gene expression of the pro-brown adipose tissue gene *Pparα* in R6/2 mice, as determined by an SAB microarray assay (PAMM‐049Z). We also found significant upregulation of the pro-brown adipose tissue genes *Ucp1* and *Zfp516*, as well as a trend towards upregulation of the pro-brown transcription factor *Creb1* and the anti-white adipose tissue gene *Gata3*. Gene expression was analyzed using the ΔΔCt method and normalized to *Gusb*, *Hsp90ab1* and *Tbp*. Bars represent mean ± SEM, * P < 0.05; ** P < 0.01, *** P < 0.001.

Validations were carried out on inguinal WAT from 12-week old R6/2 mice and WT littermates (n = 6/group). In R6/2 inguinal WAT, there was a significant upregulation of the brown adipocyte markers *Ucp1* and *Zfp516* as well as the genes *Jun*, *Sirt1*, *Pparα* and *Taz* (Fig 3).

It has been previously shown, both in HD patients and mouse models of disease, that weight loss is correlated with CAG repeat length [12]. We here investigated whether *Ucp1* gene expression in R6/2 mice correlates with body weight. Our data suggests that the gene expression levels of *Ucp1* in R6/2 mice do not correlate with decreased body weight (r = 0.28, p = 0.5897).

### Elevated UCP1 protein expression in R6/2 WAT

**Fig 4:**
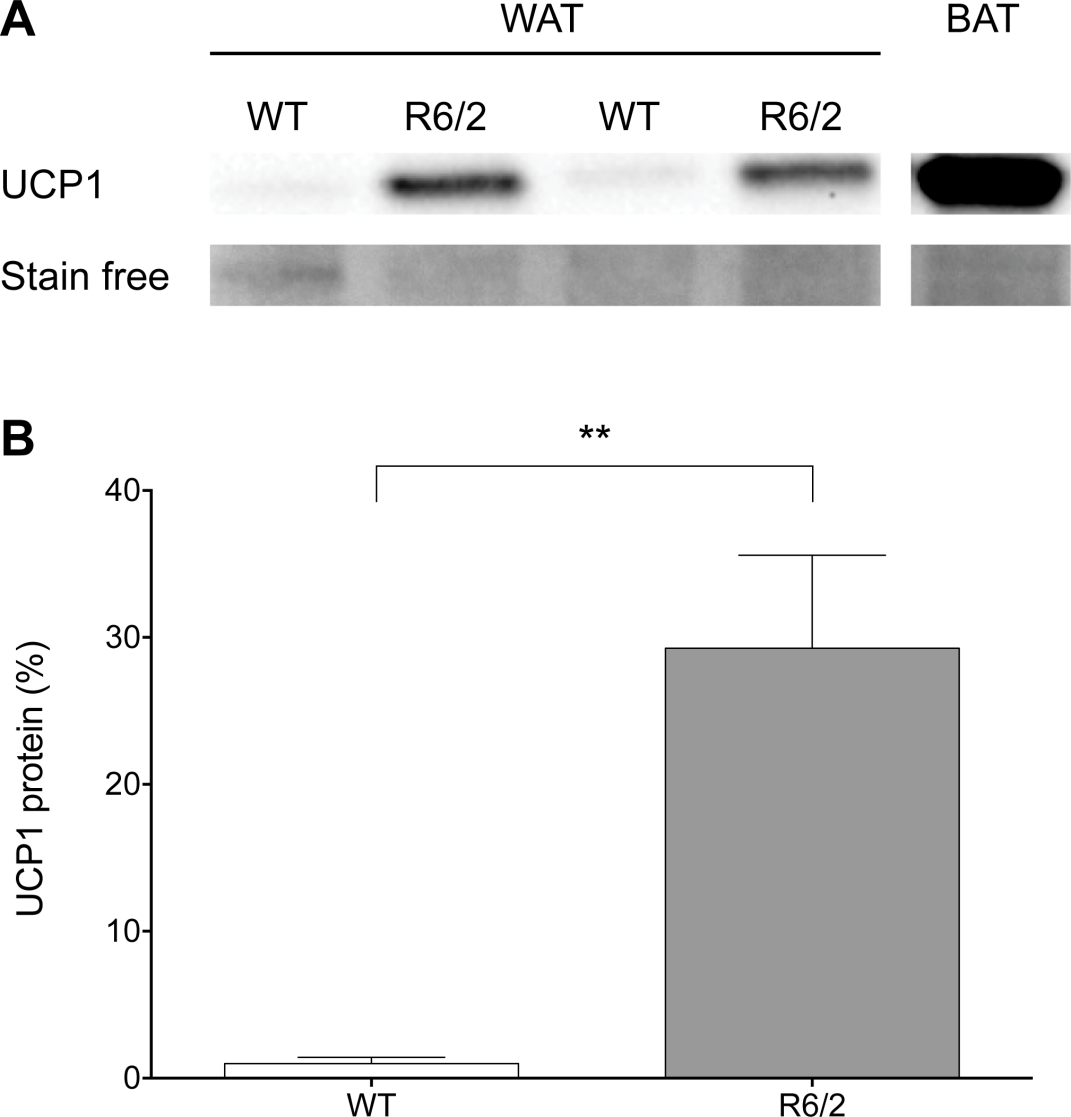
Elevated levels of UCP1 protein in R6/2 mouse inguinal WAT. Representative western blot (**A**) and densitometry (**B**) of UCP1 levels in inguinal WAT from 12-week old R6/2 and WT littermate mice. Western blot of iBAT is shown for control purposes (SFig 2). Stain-free imaging (Bio-Rad) was used as the loading control and also for normalization. Graph shows mean % above control ± S.E.M. for six mice of each genotype.

Following our RT-qPCR results indicating an upregulation of genes responsible for browning of WAT, we next determined whether this was also observed at the protein level. Western blot analysis showed that mitochondrial fractions from WT subcutaneous WAT exhibited almost undetectable levels of UCP1 protein while R6/2 samples showed a significant increase (29.27 fold increase, P = 0.0012) in UCP1 protein translation (Fig 4).

### Cold exposure-induced *Ucp1* expression is markedly increased in R6/2 inguinal WAT and blunted in R6/2 iBAT

**Fig 5:**
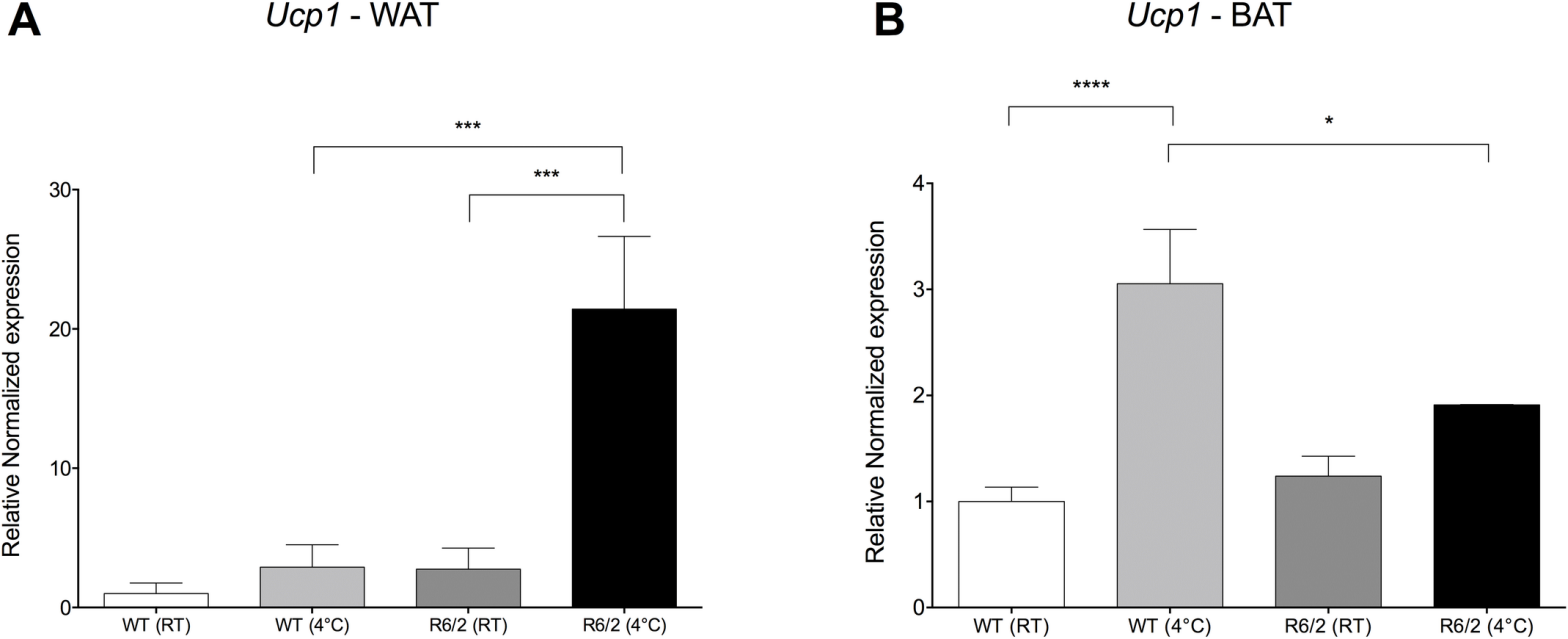
Cold exposure enhances browning of R6/2 mouse inguinal WAT. Cold exposure markedly induced *Ucp1* expression in R6/2 mouse inguinal WAT. Mice were exposed to two consecutive days of 4°C for 4 hours per day to evaluate their response to cold exposure. Normalized gene expression from 12-week R6/2 versus WT mice inguinal WAT and interscapular BAT. Bars represent WT at room temperature (white bars); WT following cold exposure (light grey bars); R6/2 at room temperature (dark grey bars); and R6/2 following cold exposure (black bars). **A** Gene expression data from subcutaneous inguinal WAT showing increased expression of brown adipose marker *Ucp1* in R6/2 WAT following cold exposure. **B** Gene expression data from interscapular BAT showing blunted expression of *Ucp1* in R6/2 iBAT following cold exposure as compared to WT. Gene expression was analyzed using the ΔΔCt method and normalized to *Gusb*, *Hsp90ab1* and *Tbp*. Bars represent mean ± SEM, * P < 0.05, *** P < 0.001, **** P < 0.0001. n = 9-10/group.

Interscapular BAT and inguinal WAT with brown features are known to undergo rapid gene expression changes in response to cold exposure [34]. Repeated cold exposure has been shown to enhance the recruitment, activity and energy expenditure of BAT, via non‐shivering thermogenesis, in both human and rodents [35–37] with an increased number of beige adipocytes within WAT [36, 37]. Therefore, we conducted a repeated cold challenge, where mice were maintained at 4°C for 4 h on two subsequent days after which fat depots were collected, and investigated the impact of repeated cold exposure on the brown marker *Ucp1* in WAT alongside BAT. Quantitative real-time PCR showed a markedly induced *Ucp1* expression in R6/2 WAT (7.38 fold increase, P < 0.001; Fig 5A) as compared to the response in WT mice following cold exposure.

Notably, in HD mice, impaired activation of iBAT, illustrated by a blunted *Ucp1* induction, was shown by Weydt and coworkers in 2006 [38]. We could here confirm a blunted iBAT *Ucp1* expression response in R6/2 mice upon cold challenge (Fig 5B). Relative to WT following cold challenge, R6/2 iBAT *Ucp1* expression is significantly reduced (-1.60 fold; P < 0.05).

### R6/2 epidiymal WAT displays altered lipolytic function

**Fig 6:**
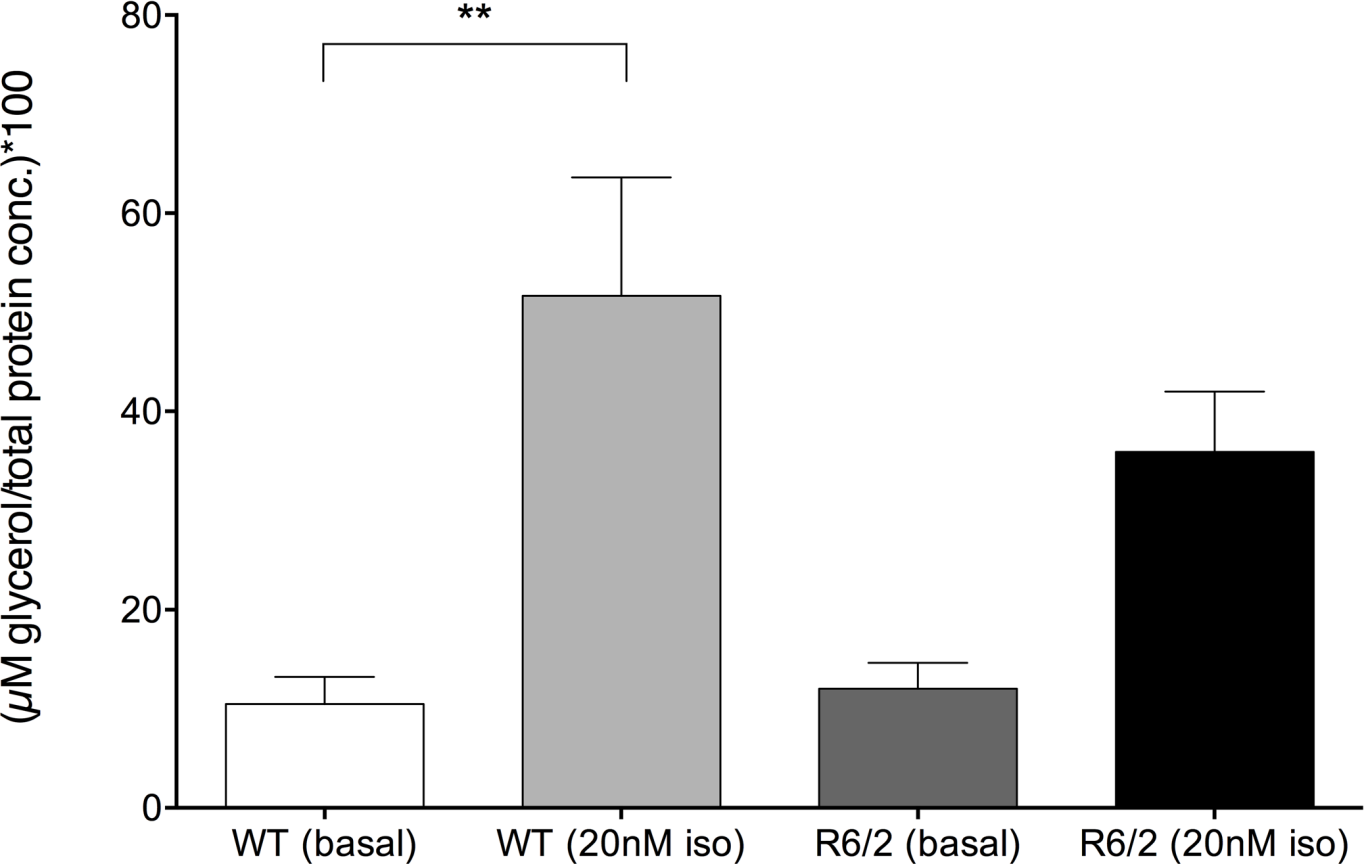
Isoprenaline-induced lipolysis is reduced in 12 week old R6/2 epididymal WAT. Glycerol concentration (relative to total protein content of cells) at baseline and following 20nM isoprenaline stimulation for 12 week old WT (white bars; n = 8–12) and R6/2 mice (grey bars; n = 9–13). Isoprenaline significantly increases lipolysis in isolated epididymal WAT of WT mice, however the response in R6/2 isolated epididymal WAT is not significant compared to basal levels. Bars represent mean ± SEM, ** P < 0.01.

Gene expression analyses (such as altered expression of *Hp*, *Pik3r1*, *Ehhadh*, *Cfd*, *Insig1*, etc.) (See S1 Table), suggest possible alterations in R6/2 WAT lipolytic function. Fain and co-workers demonstrated in 2001 that isoprenaline-stimulated liposlysis was reduced in R6/2 WAT [14], however basal lipolytic function was unaltered. Therefore, we here investigated lipolysis, as glycerol release upon isoprenaline-stimulation in fresh isolated primary adipocytes. We have in this study chosen to investigate mice at a time point at which weight loss has initiated, at this point, the mice are not end-stage. At this chosen time-point, similar to the results obtained by Fain and colleagues (Fain et. al., 2001), there was no difference in basal lipolysis, whereas the lipolytic response to isoprenaline stimulation was reduced in R6/2 epididymal WAT compared to WT WAT (Fig 6). The fold change response to isoprenaline stimulation was 4.9 in WT epididymal WAT and 3.0 in R6/2 epididymal WAT, although there was no significant difference in the lipolytic response in R6/2 mice when compared to basal levels.

### Altered oxygen consumption in R6/2 mouse subcutaneous inguinal WAT

**Fig 7:**
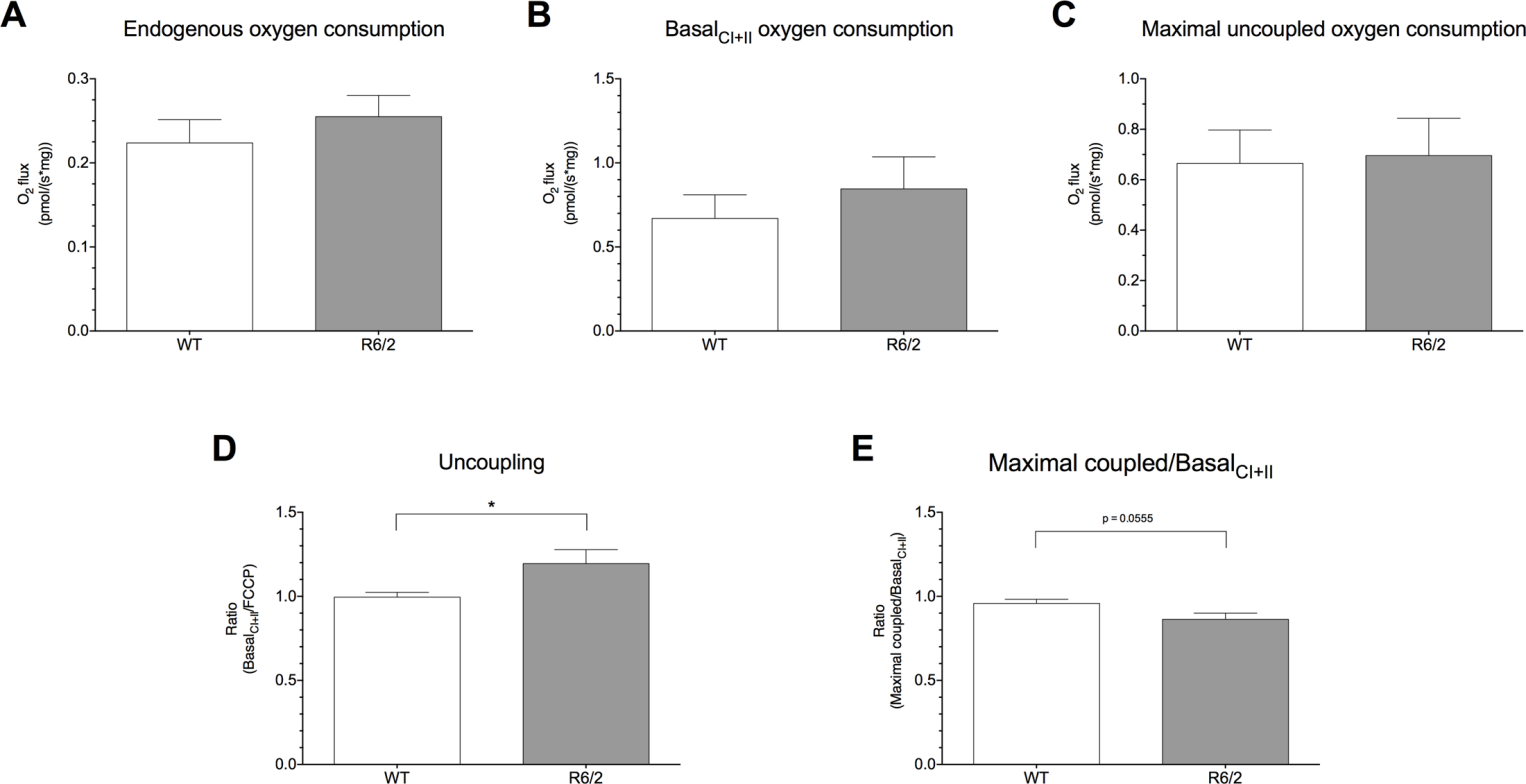
Increased oxygen consumption in R6/2 inguinal WAT. Endogenous oxygen consumption (**A**), basal_CI+II_ oxygen consumption (**B**), maximal uncoupled oxygen consumption following addition of FCCP (**C**), level of mitochondrial uncoupling (**D**) and maximal coupled/basal_CI+II_ ratio (**E**), measured using the Oxygraph-2k. Endogenous oxygen consumption and basal_CI+II_ oxygen consumption (following addition of complex I and II substrates) show subtle, but not significant, increased rates for R6/2 inguinal WAT compared to WT. Maximal uncoupled oxygen consumption following FCCP addition was comparable, however, for both genotypes. Mitochondrial uncoupling, measured as the ratio of basal_CI+II_ /maximal uncoupled oxygen consumption was significantly increased in R6/2 WAT, suggesting that the mitochondria display an increased uncoupling activity. This finding was further confirmed by a reduced maximal coupled/basal_CI+II_ ratio in R6/2 inguinal WAT. Oxygen consumption rates are expressed per mg WAT. All data are represented as means ± SEM of seven independent experiments, * P < 0.05.

Both gene and protein expression analyses revealed increased UCP1 in R6/2 WAT. To examine the functional consequences of this increased expression, we investigated oxygen consumption in isolated subcutaneous inguinal WAT from R6/2 mice by high-resolution respirometry. Compared to WT controls, R6/2 inguinal WAT shows a subtle, but not significant, increased rate of both endogenous (Fig 7A) and basal_CI+II_ (Fig 7B) oxygen consumption in the presence of complex I and II substrates. Addition of digitonin to permeabilise the cells did not result in a further increase in respiration indicating that any difference was not caused by differently permeable cell membranes in the R6/2 mice (data not shown). To determine whether the electron flux at the respiratory chain was uncoupled from oxidative phosphorylation in R6/2 inguinal WAT, we then investigated the response to the chemical uncoupler FCCP and calculated the ratio between the flux during basal_CI+II_ respiratory rate and maximal uncoupled respiration by FCCP. Here, we observed a significantly increased basal_CI+II_/FCCP ratio in R6/2 inguinal WAT compared to WT (1.19 ± 0.08 vs. 1.00 ± 0.03, P = 0.0436; Fig 7D) despite comparable respiratory rates following uncoupling with FCCP (Fig 7C), suggesting that the mitochondrial respiration in subcutaneous inguinal WAT of this HD mouse model is already uncoupled from oxidative phosphorylation. Similarly, we saw a decreased maximal coupled/basal_CI+II_ ratio in R6/2 WAT compared to WT, further suggesting enhanced uncoupling activity in R6/2 inguinal WAT (Fig 7E).

### Key brown gene expression alterations also present in Q175 mouse inguinal WAT

**Fig 8:**
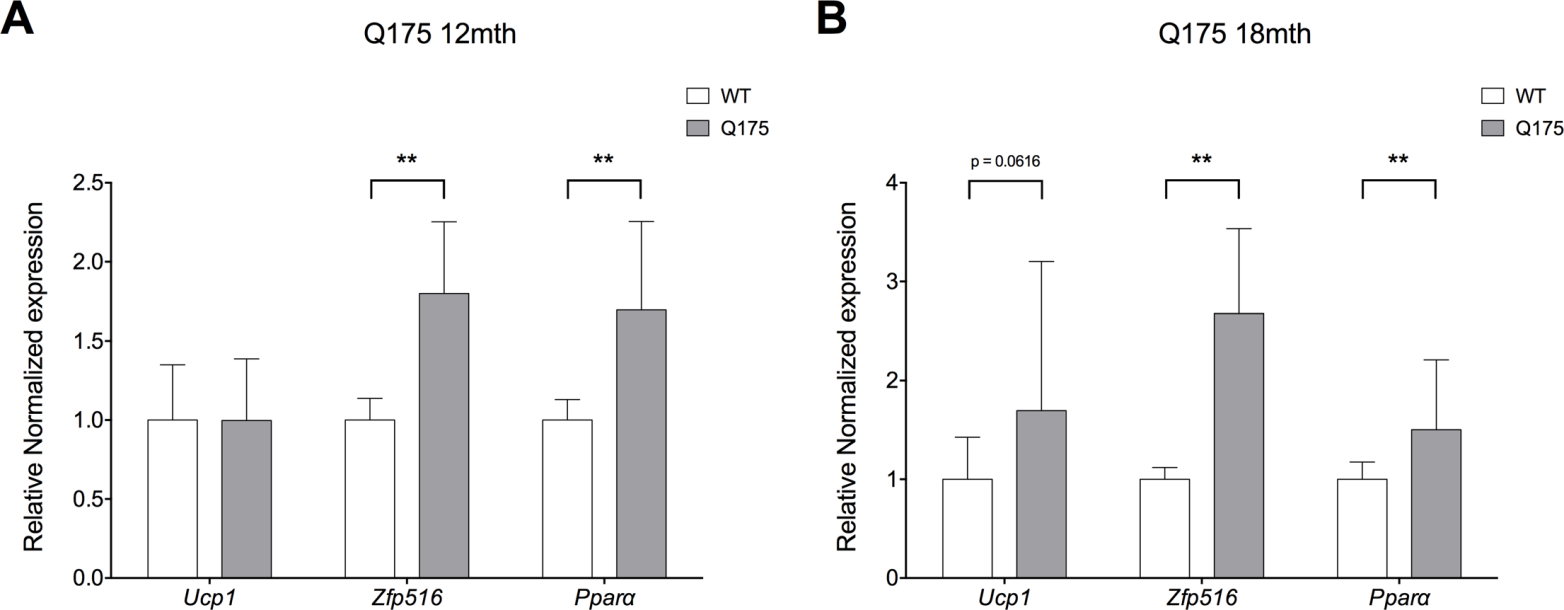
Significantly upregulated pro-brown adipose tissue gene expression in Q175 mouse inguinal WAT. Normalized gene expression from 12-month Q175 (grey bars; n = 4) versus WT (white bars; n = 6) subcutaneous inguinal WAT (**A**). Here we replicated the upregulated gene expression of the pro-brown adipose tissue genes *Pparα* and *Zfp516* as observed in 12-week R6/2 mice. Normalized gene expression from 18-month Q175 (grey bars; n = 6) versus WT (white bars; n = 6) subcutaneous inguinal WAT (**B**). Here we replicated the upregulated gene expression of the pro-brown adipose tissue genes *Pparα* and *Zfp516* as observed in both 12-week R6/2 mice and 12-month Q175 mice, and observe a trend towards upregulation of *Ucp1*. Gene expression was analyzed using the ΔΔCt method and normalized to *Gusb*, *Hsp90ab1* and *Tbp*. Bars represent mean ± SEM, ** P < 0.01.

In order to investigate whether the gene expression changes that accompany the WAT browning process found in R6/2 mice are also present in the full-length Q175 mouse model of HD, selected targets were investigated in inguinal WAT of 12- and 18-month old Q175 mice. In line with R6/2 mouse gene expression alterations, there was significantly increased expression of the pro-brown adipocyte genes *Pparα* (1.80 fold; P = 0.00353) and *Zfp516* (1.70 fold; P = 0.00570) in 12-month old Q175 mice (Fig 8A). In 18-month Q175 mice, we could similarly show significant increases in *Pparα* (1.50 fold; P = 0.00931) and *Zfp516* (2.68 fold; P = 0.00013), as well as a trend towards upregulation of *Ucp1* (1.70 fold; P = 0.06156) (Fig 8B).

## Discussion

Increased energy expenditure and a negative energy balance are largely affected by brown fat expansion and/or activation. Inducible brown-like adipocytes in white adipose tissue share the characteristics of high mitochondria content, UCP1 expression and thermogenic capacity when activated. In this study, we have found histological and gene expressional evidence for presence of brown-like adipocytes in R6/2 mouse WAT. Transcriptional changes potentially contribute to convert the fate of inguinal WAT towards brown-like. Targeted array data showed upregulation of *Pparα* and *Sirt1*. These factors have both been previously shown to mediate browning of white adipose tissue in metabolic disorders [39]. Transcriptional regulation of the *Ucp1* gene is controlled by many regulatory factors, including peroxisome proliferator-activated receptor gamma (PPARγ), C/EBP families and CREB [40]. PPAR*α* is an important regulator of energy homeostasis through its ability to stimulate fatty acid oxidation [41]. PPAR*α* is also expressed in BAT and it has been shown that PPAR*α* activators induce UCP1 expression [42]. Importantly, PPAR*α* has been shown to play a role in regulating UCP1 during induction of brown-like adipocytes in WAT [21]. In our study, *Pparα* expression is increased in both R6/2 and Q175 HD mouse inguinal WAT, indicating a possible role for the appearance of brown-like adipocytes in R6/2 WAT. Adipocyte differentiation requires complex regulation. CREB1 is a transcription factor involved in determining fate of adipocytes [21]. Potentially CREB1 plays a role in the here observed inguinal WAT browning, as CREB activation leads to downstream activation of UCP1 [43]. Interestingly, we recently showed CREB1 to be a highly significant transcription factor altered in human premanifest HD subcutaneous adipose tissue [16].

Here, we observed elevated levels of *Zfp516* mRNA in R6/2 inguinal WAT as compared to WT littermates, in line with a marked increase in *Ucp1* mRNA expression. *Zfp516* is an activator of both *Ucp1* and *Pgc1a*, thereby promoting brown adipose tissue activation [44]. This increase in *Zfp516* mRNA expression was also observed in Q175 mice at both 12- and 18-months of age.

Differentiation of adipocytes has previously been shown to be altered in HD mouse models [15]. Here, using a targeted array to investigate genes involved in the differentiation and maintenance of mature adipocytes, we found several genes to be significantly altered in R6/2 inguinal WAT (*Cfd*, *Gata3*, *Jun*, *Pparα*, *Sirt1*, *Taz* and *Wnt5b*; Table 5).

GATA-2 and -3 are transcription factors found in WAT, but not BAT, that regulate adipocyte differentiation, whereby deficiency of GATA3 enhances adipocyte differentiation and overexpression inhibits differentiation [45]. Consistent with this, we observed increased *Gata3* expression in R6/2 inguinal WAT relative to WT, indicating a reduction in white adipocyte differentiation from preadipocytes and supporting our data that R6/2 WAT undergoes browning.

A recent study highlighted miR-27 as a transcriptional regulator of brown adipogenesis [46]. Reduced miR-27 was shown to directly target key components of brown adipose transcription, such as *Pparα* and *Creb1* [46]. We have in this study demonstrated upregulation of *Pparα* and have previously highlighted the CREB1 pathway to be altered in premanifest human HD adipose tissue [16]. Possibly, since miR-27 is downregulated following prolonged cold exposure, and HD mice show progressive hypothermia [38], altered levels of miR-27 in HD mice could be involved in browning of their WAT.

In addition to classical neurological symptoms, HD is complicated by problems such as weight loss, for which the underlying mechanisms are not fully understood. Patients with early HD, as well as R6/2 mice, have been shown to display a negative energy balance [13]. Weight loss in R6/2 mice has been shown to be accompanied by increased oxygen consumption and to occur independently of increased food intake [11], indicating increased energy expenditure. White adipose tissue obtained from R6/2 mice shows evidence of inguinal and epididymal WAT browning, illustrated by histological findings (Fig 1; S1 Fig) and key gene expression alterations (Figs 2 and 3, Table 5, S1 Table). We could also show evidence of browning in inguinal WAT of the Q175 mouse model of HD (Fig 8). Taken together, this could contribute to the increased energy expenditure described to occur in HD. Indeed, our results indicate a subtle trend towards increased oxygen consumption and a significantly increased uncoupling activity in R6/2 subcutaneous inguinal WAT.

We found increased inguinal WAT expression of *Ucp1*, at both gene and protein level, along with a markedly increased inguinal WAT *Ucp1* response to cold exposure. To test whether this resulted in functional consequences and possibly higher degree of uncoupling activity in R6/2 WAT mitochondria, we utilised high-resolution respirometry to measure oxygen consumption of homogenised subcutaneous inguinal WAT from 12 week R6/2 mice and WT.

Our data show an increased uncoupled mitochondrial oxygen consumption in R6/2 inguinal WAT causing a trend towards increased endogenous and basal_CI+II_ oxygen consumption, suggesting that inguinal WAT of R6/2 mice develops characteristics of brown-like adipocytes. The appearance of brown-like adipocytes in WAT has been documented in several transgenic mouse models leading to a lean phenotype [47, 48]. It is therefore likely that a subtle increase in oxygen consumption in WAT of R6/2 mice may contribute, at least in part, to an increase in energy expenditure. HD patients and mouse models have been shown to exhibit signs of metabolic disorder, such as increased incidence of diabetes [49, 50] and cardiovascular disease [51, 52]. Increased metabolism has been previously demonstrated in the R6/2 mouse model of HD [11] and premanifest HD patients have been shown to exhibit increased energy expenditure alongside higher caloric intake and reduced BMI [53]. Further, in HD, a negative energy balance has been shown [13]; it remains possible that over time, a slight change in WAT features, such as our observation of more brown-like adipocytes, could play a part in HD-related energy metabolism and body weight balance.

In BAT, PPARγ co-activator 1alpha (Pgc-1*α*), is the key regulator of *Ucp1* expression [54]. In HD BAT, impairment of Pgc-1*α* has been shown to be linked to a blunted *Ucp1* response to cold exposure [38]. This finding we could here replicate. To our surprise, in R6/2 inguinal WAT there was however a markedly induced *Ucp1* expression response to cold exposure.

Lindenberg *et* al., using two-point magnitude MRI, showed that R6/2 mice display a 50% reduction in BAT volume as compared to WT mice [55]. The observed reduction remained significant even following normalisation to total body volume. Here, we observed a reduced *Ucp1* response in R6/2 iBAT following cold exposure (Fig 5B), suggesting that in addition to a reduction in BAT volume, R6/2 BAT also exhibits functional impairment, in line with previous findings [38]. While we observed no alteration in the expression of *Pgc-1α* in inguinal WAT of R6/2 mice, previous studies have shown its impairment in HD BAT. iBAT whitening in HD mouse models has been demonstrated [38], as well as a reduction in BAT volume [55]. Taken together, this might explain the blunted response to cold exposure observed in R6/2 mouse iBAT. This blunted response to cold exposure was not surprising, however, since HD mice display progressive hypothermia upon cold exposure [38].

Our results warrant further studies using for example MRI after cold exposure to see whether the inguinal WAT browning (Ucp1 expression) will be noted also using MRI.

Our gene expression analysis also suggests alterations in R6/2 WAT lipolytic function. Supportive of this and similar to results obtained by Fain and co-workers demonstrated in 2001 [14], we could here observe that isoprenaline-stimulated lipolysis was reduced in R6/2 epididymal WAT. Possibly, the altered fate of R6/2 mouse WAT also affects energy utilization and storage capacity. Alterations in visceral WAT have also been reported previously, with an increase in depot volume/weight with disease progression in R6/2 mice [15]. Interestingly, inflammation of visceral adipose tissue has been demonstrated to play a role in insulin resistance [56]. As insulin resistance has been shown in the R6/2 mouse model of HD [49] as well as a peripheral immune response detected in serum [57], further studies are required to elucidate whether the alterations in WAT may be linked to an inflammatory response.

In summary, our data suggests that HD mouse WAT undergoes a browning phenomenon characterised by increased *Ucp1* expression, leading to functional consequences. These changes may contribute to the weight loss and/or metabolic disturbances observed in HD. As the presence of brown-like adipocytes in WAT affect overall energy expenditure, it is highly relevant for further investigation in human HD.

## Supporting Information

S1 Fig Brown-like adipose features within epididymal WAT of R6/2 mice. Representative images of epididymal white adipose tissue (WAT) from **A** wild type (WT) and **B** R6/2 transgenic mice. Scale bars = 20 μm. **C** Representative image from R6/2 epididymal WAT showing numerous small brown-like adipocytes interspersed between larger unilocular white adipocytes. As with inguinal WAT, we failed to observe this phenomenon in the WT epididymal samples. Scale bar = 20 μm.
(TIF)

S2 Fig Uncropped Western blot. Uncropped Western blot image from which Fig 4 of UCP1 levels in inguinal WAT from 12-week old R6/2 and WT littermate mice is adapted. Stain-free imaging (Bio-Rad; right panel) was used as the loading control and also for normalization.
(TIF)

S1 Table R6/2 versus WT affymetrix data. Complete affymetrix dataset for R6/2 vs. WT inguinal WAT following SAM analysis.
(XLS)

S2 Table Transcription factor networks. Transcription factor networks involving the 69 most significantly altered genes from affymetrix analysis of R6/2 versus WT inguinal WAT.
(XLSX)

S3 Table Pathway maps. Pathway maps based on the 69 significantly altered transcripts from affymetrix analysis of R6/2 versus WT inguinal WAT.
(XLSX)
