# Supplementary figures and images for: White Adipose Tissue Browning in the R6/2 Mouse Model of Huntington’s Disease
Source: PLoS One. 2016 Aug 3;11(8):e0159870. doi: 10.1371/journal.pone.0159870 (PMC4972251; doi:10.1371/journal.pone.0159870)

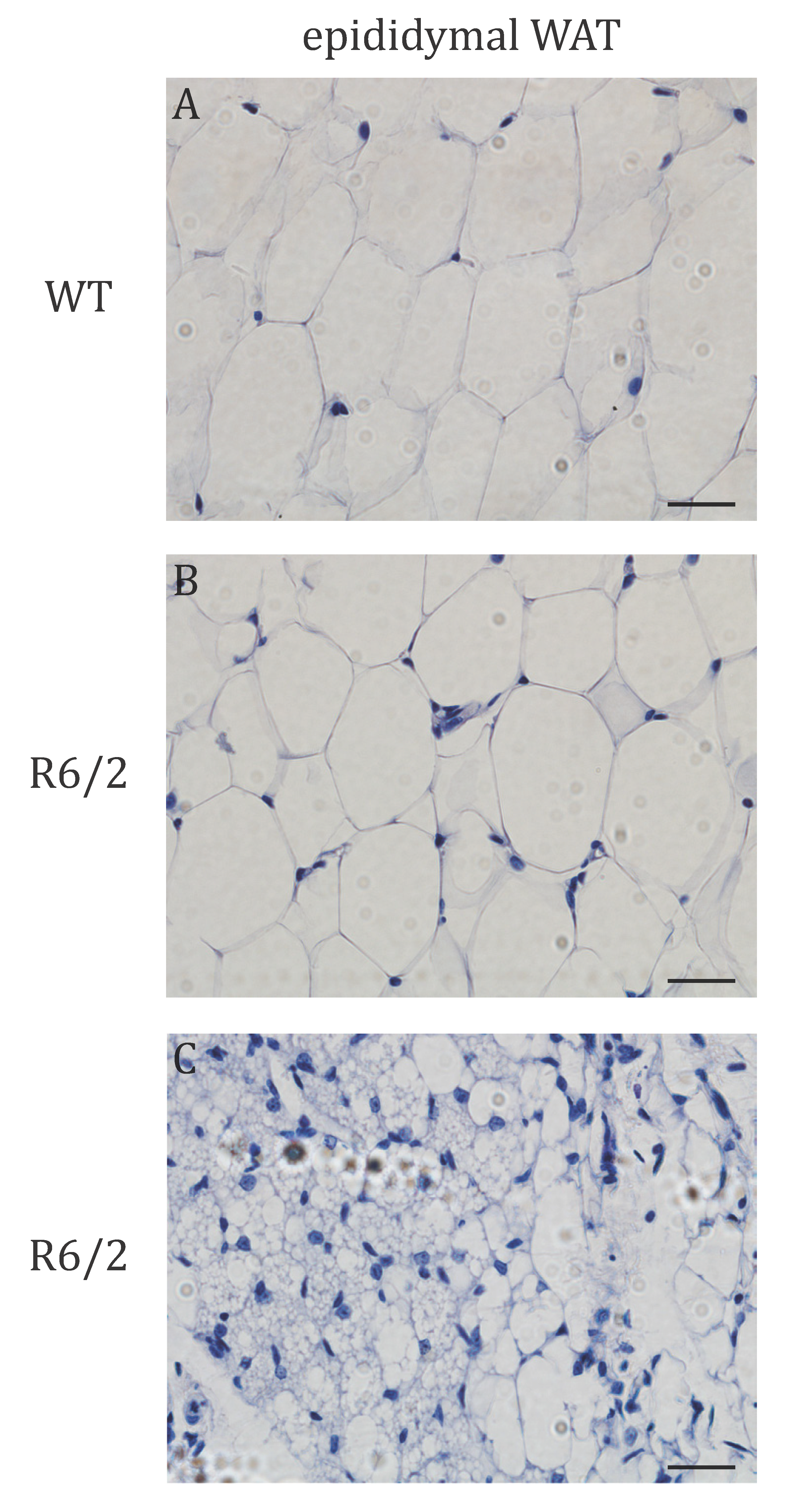

Supplement: S1 Fig — Representative images of epididymal white adipose tissue (WAT) from A wild type (WT) and B R6/2 transgenic mice. Scale bars = 20 μm. C Representative image from R6/2 epididymal WAT showing numerous small brown-like adipocytes interspersed between larger unilocular white adipocytes. As with inguinal WAT, we failed to observe this phenomenon in the WT epididymal samples. Scale bar = 20 μm. (TIF) [file pone.0159870.s001.tif]

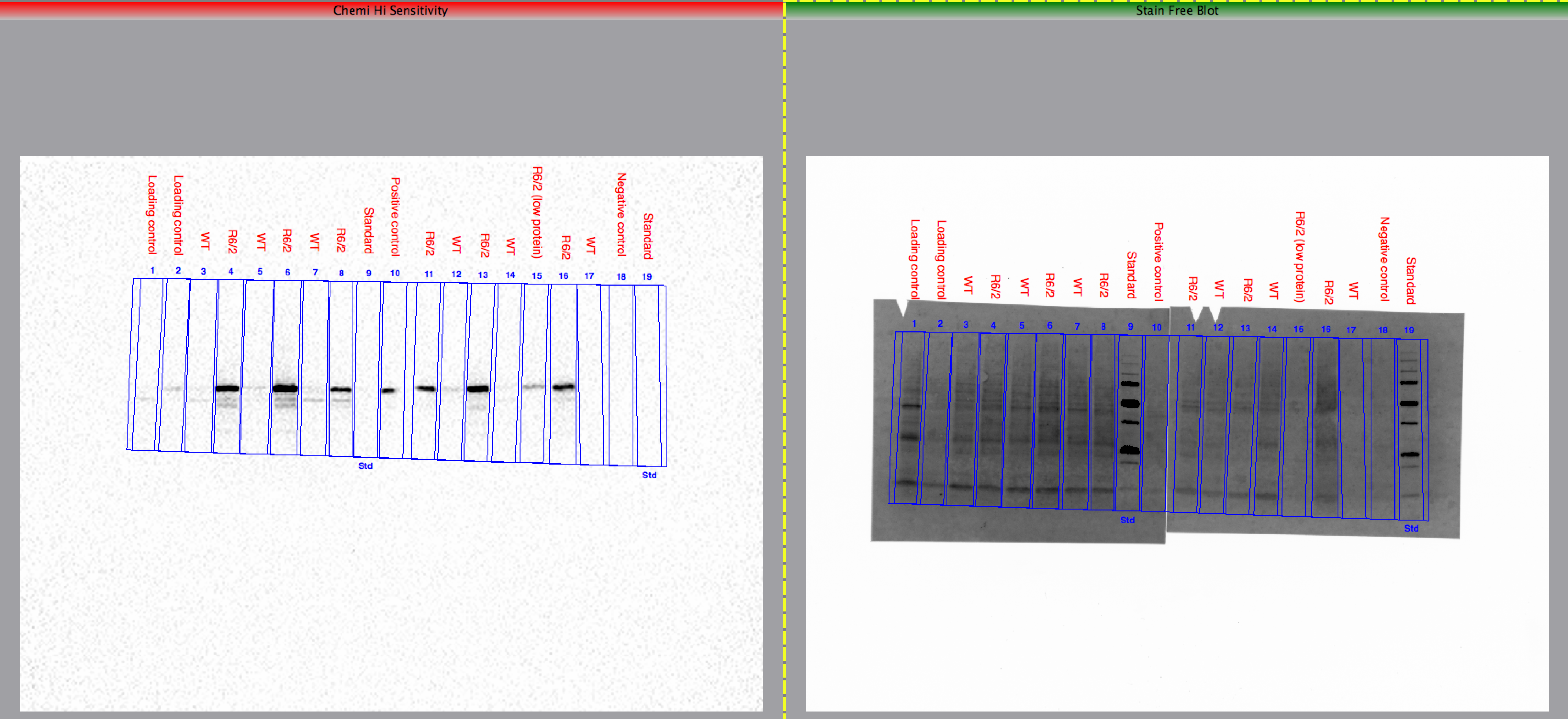

Supplement: S2 Fig — Uncropped Western blot image from which Fig 4 of UCP1 levels in inguinal WAT from 12-week old R6/2 and WT littermate mice is adapted. Stain-free imaging (Bio-Rad; right panel) was used as the loading control and also for normalization. (TIF) [file pone.0159870.s002.tif]
